# European Lobster Larval Development and Fitness Under a Temperature Gradient and Ocean Acidification

**DOI:** 10.3389/fphys.2022.809929

**Published:** 2022-07-14

**Authors:** Laura Leiva, Nelly Tremblay, Gabriela Torres, Maarten Boersma, Roland Krone, Luis Giménez

**Affiliations:** ^1^ Alfred-Wegener-Institut Helmholtz-Zentrum für Polar- und Meeresforschung, Biologische Anstalt Helgoland, Helgoland, Germany; ^2^ FB2, University of Bremen, Bremen, Germany; ^3^ Reefauna - Spezialisten für Rifftiere, Bremerhaven, Germany; ^4^ School of Ocean Sciences, College of Environmental Sciences and Engineering, Bangor University, Menai Bridge, United Kingdom

**Keywords:** climate change, ocean warming, thermal tolerance, early life stages, decapod

## Abstract

Climate change combined with anthropogenic stressors (e.g. overfishing, habitat destruction) may have particularly strong effects on threatened populations of coastal invertebrates. The collapse of the population of European lobster (*Homarus gammarus*) around Helgoland constitutes a good example and prompted a large-scale restocking program. The question arises if recruitment of remaining natural individuals and program-released specimens could be stunted by ongoing climate change. We examined the joint effect of ocean warming and acidification on survival, development, morphology, energy metabolism and enzymatic antioxidant activity of the larval stages of the European lobster. Larvae from four independent hatches were reared from stage I to III under a gradient of 10 seawater temperatures (13–24°C) combined with moderate (∼470 µatm) and elevated (∼1160 µatm) seawater *p*CO_2_ treatments. Those treatments correspond to the shared socio-economic pathways (SSP), SSP1-2.6 and SSP5-8.5 (i.e. the low and the very high greenhouse gas emissions respectively) projected for 2100 by the Intergovernmental Panel on Climate Change. Larvae under the elevated *p*CO_2_ treatment had not only lower survival rates, but also significantly smaller rostrum length. However, temperature was the main driver of energy demands with increased oxygen consumption rates and elemental C:N ratio towards warmer temperatures, with a reducing effect on development time. Using this large temperature gradient, we provide a more precise insight on the aerobic thermal window trade-offs of lobster larvae and whether exposure to the worst hypercapnia scenario may narrow it. This may have repercussions on the recruitment of the remaining natural and program-released specimens and thus, in the enhancement success of future lobster stocks.

## Introduction

Since the industrial age, the burning of fossil fuels has led to an exponential increase in CO_2_ emissions and temperature. To understand the impact of climate change and potential risks, future scenarios have been developed by the Intergovernmental Panel of Climate Change (IPCC), which are based on diverse degrees of mitigation efforts to decrease CO_2_ emissions. The low mitigation scenario SSP5-8.5, predicts that by the end of this century, sea surface temperature (SST) will have increased by 2.58°C, seawater surface pH will have decreased to 7.7; and atmospheric CO_2_ could have reached 1000 µatm. In contrast, the high mitigation scenario SSP1-2.6 corresponding mean changes are +0.73°C SST, reduction to pH 8.0 for surface seawater, and approximately 430–480 µatm CO_2_ concentrations (IPCC, 2014; Pörtner et al., 2019; IPCC, 2021).

The combined effects of ocean acidification (OA) and warming on marine life have been studied for at least two decades, but they are still challenging to interpret and predict. A growing number of experiments using ecologically and economically important species, such as Pacific herring (Villalobos et al., 2020), Pacific oysters (Lemasson et al., 2018), gilthead seabream, meagre (Pimentel et al., 2016), American and European lobster (Small et al., 2015; Waller et al., 2017) have shown an exacerbated impact of OA on survival, physiology, and growth when it was combined with elevated temperatures. Synergistic impacts (the result of stressors interacting and producing a greater effect than the cumulative or individual effects) of climate change vary across life stages with the tendency that early life stages are more sensitive and less tolerant to environmental stressors than adults (Kikkawa et al., 2003; Ishimatsu et al., 2004; Kurihara, 2008). Understanding the synergistic effects of OA and warming on larval development is critical to predict how climate change will influence larval survival, dispersal and hence, population connectivity (Cowen and Sponaugle, 2009; Giménez et al., 2020). This is particularly important for the future of commercially important and vulnerable species, like crustaceans, which have complex life cycles and undergo distinct ontogenetical changes. As in the majority of marine species with planktonic larvae, the transition between the larval pelagic stage to the benthic post-larval stage larvae has been described as a population bottleneck (Marshall and Morgan, 2011).

European lobster (*Homarus gammarus*) develops through three pelagic larval stages (stages I, II and III), a postlarval stage (stage IV) and then reaches the juvenile stage which marks the complete transition to a benthic lifestyle (Charmantier et al., 1991). The International Union for Conservation of Nature has listed the European lobster as “least concern” as the examination concluded that this species has a broad geographic range, despite commercial fisheries. This is perhaps true on a global level, but not for the lobsters of the German Bight, North Sea, that inhabit the rocky shores of the island of Helgoland. This population experienced a dramatic decline in the 1950s and 1960s from a combination of overfishing, pollution and extensive habitat destruction (Franke and Gutow, 2004). The decline of this population prompted a large-scale restocking program on Helgoland, presently carried out by the lobster conservation company, Reefauna. After 10 years (1999 – 2009) of releasing hatchery-reared juveniles into the wild, the success of the restocking program was evaluated; the results showed that re-stocked lobsters could be re-caught; survival rates averaged 40% and the proportion of caught cultured lobsters to wild lobsters was 3–8% between the years 2007–2009 (Schmalenbach et al., 2011). Nevertheless, despite recapture of marked lobsters, it is unknown whether recruitment is successful. Most of the catches were older individuals and lobster larvae are rarely caught in long-term plankton net monitoring around the island (Greve et al., 2004). Therefore, the question arises if recruitment of remaining natural individuals and program-released specimens is currently successful, or could be affected by ongoing climate change (i.e. ocean warming and acidification).

Only two studies have assessed the joint effects of OA and ocean warming on lobster larvae of the genus *Homarus*. They provide the first insight on how lobsters may respond to the synergistic effects of environmental changes predicted for the end of the 21st century (Small et al., 2015; Waller et al., 2017). These studies have in common an experimental design based on only two temperatures and two *p*CO_2_ regimes, comparing (in a factorial design) ambient temperature and *p*CO_2_ conditions with increased temperature and *p*CO_2_. Both studies demonstrated that elevated temperature has a stronger effect on life history (survival and development) and physiological responses (oxygen consumption rates) of lobster larvae than elevated *p*CO_2_. Nevertheless, it remains unknown how lobsters will react to a broader range of temperatures under ocean acidification. Regional differences from the global mean SST and CO_2_ uptake trends can result in a “temperature buffering” effect, possibly mitigating some of the negative impacts of OA. Therefore, as suggested by Humphreys (2017), OA experimental setups should be combined with a thermal gradient to reflect regional variation from the global mean SST more realistically.

How marine crustaceans will perform under future high CO_2_ can be interpreted by their physiological capacities to adjust to environmental change. Crustaceans are water breathers and are directly exposed to ocean acidification through their gills which are specialized for respiratory gas and ion exchange (Taylor and Taylor, 1992). An acute rise in seawater *p*CO_2_ reduces (or reverses) the *p*CO_2_ diffusion gradient across the gills, causing additional CO_2_ to accumulate in the haemolymph (extracellular compartment) until an excretory gradient is restored (Whiteley, 2011). Regulating haemolymph pH is necessary to maintain proper oxygen supply, when the concentration of CO_2_ in the haemolymph increases and pH decreases it causes hemocyanin (oxygen transporting proteins) to release their load of oxygen molecules as explained by the Bohr effect (Hirota et al., 2008; Strobel et al., 2012). Nonetheless, crustaceans are equipped to buffer changes in haemolymph pH to some extent through iono-regulation (Whiteley et al., 1999; Whiteley, 2011). However, acute and long-term exposure to OA could interrupt this acid-base equilibrium and alter metabolism and growth (Whiteley and Taylor, 1992; Whiteley et al., 1999). Thus, routine metabolic rate (RMR) is traditionally investigated in studies on multiple environmental stressors as an approach to assess if elevated CO_2_ concentrations affect the sensitivity of organisms to thermal extremes (Storch et al., 2011; Waller et al., 2017; Laubenstein et al., 2019). At optimal temperatures, organisms have maximal aerobic capacity and proper functioning (Pörtner, 2001). While at suboptimal temperatures aerobic capacity is limited and failure to sustain a balance between metabolism, development and growth can result in reduced body mass at critical life history stages (Anger, 2001; Pörtner, 2008; Torres and Giménez, 2020). The suboptimal temperatures can be divided into the *pejus* range, where an organism performance starts to decrease (Frederich and Pörtner, 2000; Frederich et al., 2009); and the *pessimus* limit, when an organism switches from aerobic into anaerobic metabolism (Jost et al., 2012). Additionally, the energetic costs of maintaining proper functioning under increased *p*CO_2_ levels can further interrupt defense mechanisms against reactive oxygen species (ROS), leading to oxidative stress and lipid peroxidation (Rato et al., 2017). Therefore, the decrease in antioxidant response and consequent lipids’ oxidative damage can serve as a proxy to identify when an organism’s defense mechanism has been compromised or suppressed by environmental stressors (Beliaeff and Burgeot, 2002; Rato et al., 2017; Tremblay et al., 2020).

The purpose of our study was to provide a more complete picture on how European lobster larvae will perform in future CO_2_ conditions by including a wider range of temperature treatments. Our study investigated the ability of European lobster larvae to survive and develop successfully as well as their aerobic metabolic capacity when exposed to the projected SPP1- 2.6 scenario OA conditions and a temperature range that covers cold and warm suboptimal temperatures. Larvae from four independent hatches were reared from stage I to III under a gradient of 10 different seawater temperatures (13–24°C) combined with moderate (average ∼470 µatm) and elevated (average ∼1160 µatm) seawater *p*CO_2_ treatments (corresponding to the very stringent [SSP1-2.6] and worst-case emission scenario [SSP5-8.5] projected for 2100 by IPCC).

## Materials and Methods

### Animal Collection and Maintenance

The study was carried out at AWI Helmholtz-Zentrum für Polar-und Meeresforschung (Helgoland, Germany). The experiment was repeated four times under the same temperature and light regimes (12:12 h light/dark), each experimental run was carried out with larvae from different females or hatches, hereafter referred to hatches. Hatches typically vary due to genetic or maternal effects. Thus, pooling larvae together from different females could potentially mask responses to the treatments. This is the reason why we chose to repeat the experiment with four independent hatches to increase the robustness of the results. European lobster larvae (*Homarus gammarus)* hatched during summer from four ovigerous female lobsters captured by local fishermen in the rocky subtidal zone around the island of Helgoland (German Bight, North Sea, 54°11:3′N, 7°54.0′E). Females were fed by the lobster conservation company Reefauna and kept in separate tanks (29 × 79 cm), filled with running seawater from the North Sea under a natural light cycle until hatching occurred. Freshly hatched larvae were transferred to 60 × 800 ml glass beakers and were distributed evenly into four sections to minimize cannibalism. Two 100 cm^2^ plastic meshes (mesh size: 500 µm) were sewn in the middle and placed in the beaker to delimit these areas. The number of larvae was 15 per beaker for the first hatch and was adjusted to 12 afterwards for better survival. In total 2,880 lobster larvae were used. The research presented in this paper complies with the guidelines from the directives 2010/63/EU of the European parliament and of the Council of 22nd September 2010 and the German law on the protection of animals used for scientific purposes.

### Experimental Design and Seawater Parameters

A thermal gradient incubator similar to the one used by Thomas et al. (1963) was used for the experimental setup. The table was built to hold 60 beakers (10 columns × 6 rows) and connected to two cooling bath thermostats with engine coolant flowing through a closed system (HUBER Compatible Control K6, Offenburg, Germany) that were set at 11.8 and 27.8°C. A gradient of 10 temperatures was obtained in the horizontal axis (Supplementary Table S1). On the vertical axis, two CO_2_ concentrations were set at target 450 µatm (SSP1-2.6 scenario) and 1150 µatm (SSP5-8.5 scenario) and supplied with gentle bubbling in three rows for a total of 30 beakers per CO_2_ concentration. Each beaker had a plastic hose with a glass tube extremity connected to a CO_2_ distributor. The targeted CO_2_ levels were reached using a system that removes CO_2_ from ambient air with a soda lime filter. The CO_2_-free air (<1 µatm CO_2_) was mixed with pure CO_2_ (Air Liquide Deutschland ltd., Düsseldorf, Germany), and the *p*CO_2_ of the mixture was continuously monitored with a gas detection unit (GDZ 401, Umsitec, Denkendorf, Germany) that automatically adjusts the CO_2_ concentration and flow rates to maintain the target values. All beakers were covered by a clear plastic bag to limit CO_2_ outgassing throughout the experiments. The setup resulted in a triplicate per temperature and CO_2_ concentration (*see* Supplementary Table S1). Lobster larvae were raised from stage I to stage III under the temperature and *p*CO_2_ conditions related to their position in the gradient table. Larvae were fed *ad libitum* (ca. 200–300 *Artemia salina* nauplii) after the daily water change at 9:00.

Seawater parameters were measured daily (*n* = 1 for each combination of temperature CO_2_ concentration), using a pH meter (WTW pH315i, Wilheim, Germany) and pH electrode (WTW SenTix 21 Basis pH-combined electrode, Wilheim, Germany), salinometer (WTW Cond 3110 SET 1, Wilheim, Germany) and salinity sensor (WTW, Conductivity Cells TetraCon), and thermometer (VOLTCRAFT DET2R, Wernberg-Köblitz, Germany) (*see* Supplementary Table S1). Total alkalinity (TA) was measured at the beginning and end of all experimental runs (*n* = 2 for each combination of temperature CO_2_ concentration). For TA, water was sampled airtight in 100 ml bottles and stored at 4°C until later measurements with a TitroLine α plus titrator (SI Analytics GmbH [Xylem], Weilheim, Germany) in technical duplicates with Dickson Batch 104 (NOAA, Reference material for oceanic CO_2_ measurements, 2010) as a standard. The seawater carbonate system was calculated based on measured TA, temperature, pH, salinity and pressure using the CO_2_SYS Excel Macro software (Pierrot et al., 2006). The following calculations were used, Mehrbach et al. (1973) refitted by Dickson and Millero (1987) for the CO_2_ constant, total scale (mol/kg-SW) for pH scale, Uppström (1974) for total boron and Dickson (1990) for KHSO_4_ to calculate the carbonate system. The obtained values are summarized in Supplementary Table S2, the mean values of *p*CO_2_ treatments among all temperatures were 467 ± 19 for the moderate *p*CO_2_ treatment and 1156 ± 27 for the high *p*CO_2_ treatment.

### Survival and Development Time

Lobster larvae in each beaker were monitored during the daily water change to record mortality and dead larvae were removed immediately. Cumulative survival was expressed as the percentage of the number of larvae introduced into each beaker at the start of the experimental run. To monitor development, beakers were checked daily at 9:00 for evidence of molting. Larvae were individually observed for stage characteristics, such as the formation of pleopods for stage II and the formation of uropods for stage III. When larvae molted to stage III, they were removed from the beaker for further measurements (*see* next sections). Sampling was divided into three groups once larvae reached stage III: *1*) three larvae from each beaker were sampled for RMR and afterwards frozen for biomass, carbon and nitrogen measurements; *2*) three larvae per beaker were photographed for size and morphology analysis and *3*) three larvae from each beaker were immediately frozen for enzymatic antioxidants analysis. Each experimental run lasted approximately 28 days, to allow all larvae in different temperatures treatments to reach stage III.

### Routine Metabolic Rate (RMR) Measurements

RMR was used as a proxy to investigate the effect of elevated *p*CO_2_ and temperature on stage III larvae metabolism. RMR measurements were done under the corresponding experimental temperature and freshly prepared *p*CO_2_ conditioned seawater. To make sure larvae were in a post-absorptive state, larvae were starved for 2 h to allow gastric processing (Kurmaly et al., 1990; McGaw and Curtis, 2013) in 20 ml glass vials implemented with an optically isolated oxygen sensor type PSt5 at its bottom (PreSens, Regensburg, Germany). During this 2 h, vials were covered with a mesh to avoid larvae escape and permit oxygen diffusion in the conditioned seawater. This period also allowed larvae to recover from handling stress. After 2 h, vials were tightly closed with a plastic lid, while submerged in the corresponding conditioned seawater in order to avoid air bubbles and placed on a SDR SensorDish^®^ Reader (PreSens, Regensburg, Germany). This system consists of a 24-channel reader of oxygen luminescence quenching and provides a high-quality measurement without oxygen consumption or gas exchange between the environment and the vial functioning as the incubation chamber. The system was calibrated at each temperature with seawater at 100 and 0% air saturation following the manufacturer’s protocol. A 12-well microplate was adapted to the system to measure simultaneously 12 glass vials (20 ml). Vials without larvae (*n* = 2) were used as a control to account for microbial oxygen consumption. Vials and channel readers were placed on a rocking platform shaker (IKA Rocker 2D digital, Staufen, Germany) at 80 revolutions per minute (rpm) to avoid oxygen stratification within the vials during measurement. The vials were incubated in the dark with an opaque black plastic box. The oxygen concentration was recorded every 15 s during 4 h. Oxygen levels during measurement were monitored closely to avoid suboptimal levels (<4 mg•L^−1^) inside the chambers. Oxygen consumption was determined by a linear regression of the change in O_2_ concentration data plotted against time. After RMR was measured larvae were frozen for further biomass, carbon and nitrogen measurements (*see* next section) to express RMR in O_2_ mg•h^−1^•mg DM^−1^. RMR was measured in postmolt larvae to allow comparison at all temperature treatments, as the intermolt period of larvae is greatly dependent on temperature and is thus highly variable. Past studies measuring RMR in lobster larvae show respiration rates are fairly consistent between intermolt and postmolt stage III larvae (Sasaki et al., 2011).

### Biomass, Carbon and Nitrogen Content

Freshly molted stage III larvae used for RMR were sampled for dry body mass, and carbon/nitrogen content measurements. Carbon was measured as a proxy for reserves (lipid content) and nitrogen as a proxy for protein content. The same parameters were measured in freshly hatched larvae (8–15 replicates per hatch) (*see* Supplementary Table S3). Larvae were rinsed gently with distilled water, blotted dry to remove salts and excess water and stored in 1.5 ml microcentrifuge tubes at −20°C for later analysis. For the analysis, larvae were placed in pre-weighed zinc cartridges (8 × 11 mm, LabNeed, Germany), then freeze-dried for 48 h (Christ Alpha 1–4 freeze dryer, Germany) and afterwards weighed to the nearest 0.0001-mg using a microbalance (Sartorius SC2, Germany). Carbon and nitrogen contents were then measured using an element analyzer (vario MICRO cube CHNS analyzer, Elementar Analysensysteme, Germany).

Dry mass (DM) was measured in freshly hatched larvae (8–15 replicates per hatch) to calculate instantaneous growth. Instantaneous growth rate was calculated as:

g=log(DMfDM0)T

In this formula $DM_{f}$ is the corresponding dry mass value at stage III, $DM_{0}$ is the dry mass value at hatching, and T is the development time from hatching to stage III. Total production was calculated as an additional parameter to investigate fitness of larvae and was calculated as the number of survivors to stage III in each treatment multiplied by the corresponding dry mass.

### Morphological Measurements

As a proxy to assess possible malformation under high *p*CO_2_, as seen in a study on the effect of OA on lobsters (Agnalt et al., 2013), we measured eight morphological traits. Stage III larvae were placed laterally in a Petri dish and photographed using an Olympus SZX16 stereo microscope. Pictures were then analyzed using ImageJ Software (ImageJ 1.45s, National Institute of Health, Madison, WI, United States). Eight morphological characteristics were measured following the protocol of a similar study in American lobsters *(Homarus americanus)* (Menu-Courey et al., 2019): *1*) rostrum length (RL), *2*) carapace length (CL), *3*) total length (TL), *4*) telson length, *5*) the dominant claw pollex, *6*) the dactylus, *7*) the eye diameter, which consisted of measuring the dark area. The abdomen length was calculated as the difference between TL and the sum of RL and CL.

### Antioxidant Enzyme Activity

Stage III larvae were sampled and immediately snap-frozen in liquid N_2_ and kept at −80°C until analyzed. To determine the level of cellular stress larvae experienced under experimental conditions and the mechanisms involved in the response, four antioxidant enzymes were analyzed in technical triplicates: superoxide dismutase (SOD), glutathione S-transferase (GST), glutathione peroxidase (GPx) and catalase (CAT). Each individual was cut into two pieces below the carapace and ground in liquid N_2_ using a ceramic pestle. The front part (carapace) was used for antioxidant enzymes. We aimed to quantify lipid damage using the abdomen part of the larva *via* malondialdehyde (MDA) formation, but these data were discarded as they were mostly under the detection level. For the enzymes’ analysis, the samples were transferred to microcentrifuge tubes with 125 µl of phosphate buffer solution [50 mM potassium phosphate dibasic and monobasic mixture (K_2_HPO_4_/KH_2_PO_4_, 30.5 and 19.5% respectively), 50 mM Ethylenediaminetetraacetic acid (EDTA), 1 mM phenylmethanesulphonyl fluoride, pH 7.5], homogenised using a laboratory ball mill (MIXER MILL MM 400, Retsch, Haan, Germany) and centrifuged at 23,897 *g* for 3 min at 4°C to obtain the supernatant used for the assays. SOD catalyses the conversion of O_2_•^−^ to H_2_O_2_ and was measured using xanthine-xanthine oxidase as a superoxide radical generating system and nitroblue tetrazolium as a detector (Suzuki et al., 2000). GST modifies xenobiotics into other conjugates using reduced glutathione (GSH) as substrate, and was estimated by detecting the formation of the thioether product from the reaction between GSH and 1-chloro-2,4-dinitrobenzene (Habig and Jakoby, 1981). GPx removes H_2_O_2_ using nicotinamide adenine dinucleotide phosphate (NADPH) as substrate and was measured by monitoring the decrease in the concentration of NADPH at 340 nm upon addition of H_2_O_2_ to the assay mixture (Ahmad and Pardini, 1988). CAT eliminates H_2_O_2_ too and prevents its accumulation in cells and tissues. The decrease of the H_2_O_2_ concentration catalyzed by CAT was measured at 240 nm according to Aebi, (1984). Soluble protein was also measured as per Bradford (1976) in all supernatants to obtain enzyme activities expressed in activity units (U)•mg protein^−1^. All spectrophotometric measurements were done at room temperature (20°C) using a spectrophotometer (THERMO Multiskan Spectrum, Waltham, United States).

### Data Analysis

After data visualization, statistical analyses of the defined variables were performed in RStudio Team (2021). Generalized additive models (GAM) with random effects using the package *mgcv* (Wood, 2017) were done with temperature and CO_2_ concentration as fixed factors, plus the addition of the hatch as a random factor (specified as: *s(hatch, bs= “re”)*) for all measured variables: survival, development time, morphological measurements, RMR, biomass, and antioxidant enzyme activities. The best model (interactive, additive, temperature and CO_2_ only, null model) was then chosen based on the Akaike information criterion (AIC) score and simplicity of the model (Supplementary Table S4). Line graphs were plotted using the smoothing command from the package *mgcv* (Wood, 2017) with the predicted regression line in *ggplot2 (* Wickham*,* 2016 *)*. The lines are the predicted regression lines: solid black lines were plotted when there was a temperature effect, but no effects of OA. Red and blue lines were plotted when there was a temperature and CO_2_ additive effect; the red and blue dots represent each sampled larva under high or moderate *p*CO_2_ conditions respectively. Additionally, a multivariate analysis using a principal component analysis (PCoA) was used to visualize morphological measurements by *p*CO_2_ and temperature treatments and permutational multivariate analysis of variance (PERMANOVA) to test significance.

## Results

We did not observe any evidence of a synergistic effect of high temperature and high *p*CO_2_ in any of the studied variables. Statistically this means that in no case the model including interactions between temperature and *p*CO_2_ concentration provided a better fit to the data than the models with the two main factors alone. In general, most variables were affected by temperature and effects of *p*CO_2_ (when present) were additive with respect to temperature. We present our results as the average response of larvae under each experimental condition (*p*CO_2_ and temperature) and hatch for better visualization. For results separated by hatch *see* Supplementary Figures S1–S5.

### Survival and Development Time

**FIGURE 1:**
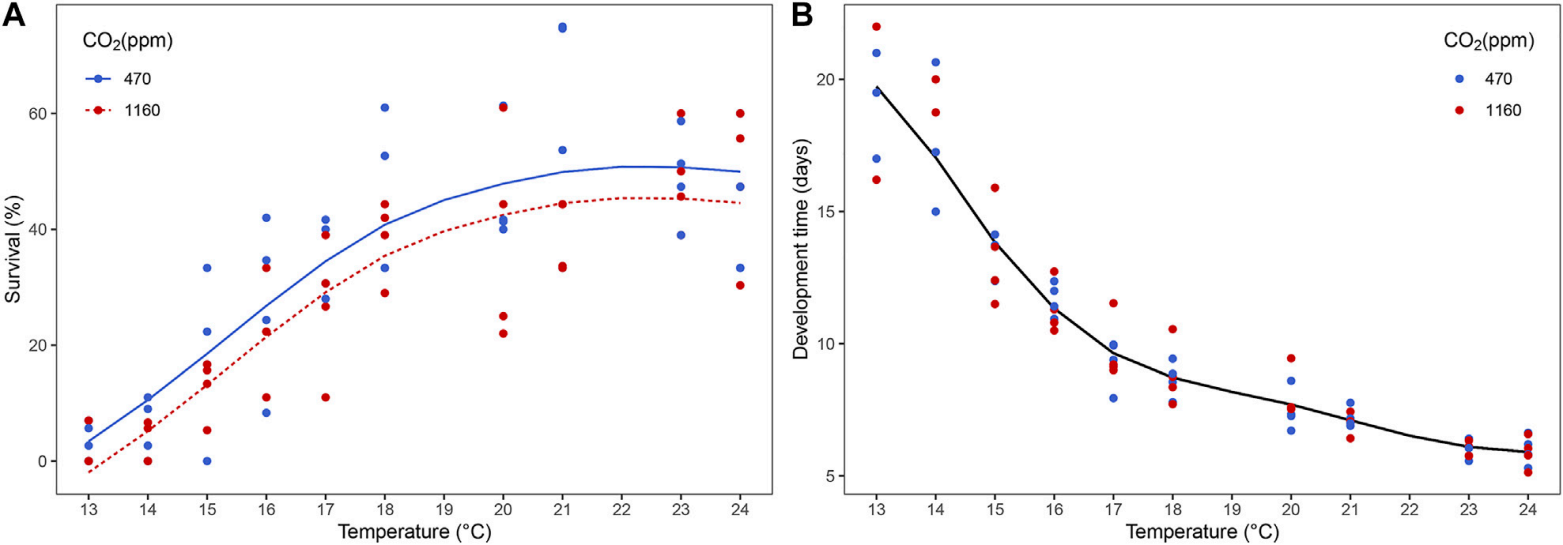
Effect of temperature and ocean acidification on the survival and development time of *Homarus gammarus* larvae to stage III. **(A)** survival was influenced by CO_2_ and temperature, **(B)** development time was influenced by temperature. Each point represents the average response quantified in larvae originated from the same female. Curves correspond to smooths fitted with the best (general additive) model, obtained after backwards model selection.

We observed evidence of a negative effect of high *p*CO_2_ on larval survival (Supplementary Table S4) but not on development time to reach stage III. At higher temperatures, the differences in average survival between CO_2_ treatments were small compared to lower temperatures. However, the best model did not retain a term indicating that smooths are conditional on the CO_2_ level. Overall, mean survival for all temperatures under moderate *p*CO_2_ was 33.3% in comparison to 27.9% for high *p*CO_2_. Moreover, survival increased with temperature while duration of development time to reach stage III decreased (Figure 1).

### Biomass and Carbon and Nitrogen Content

**FIGURE 2:**
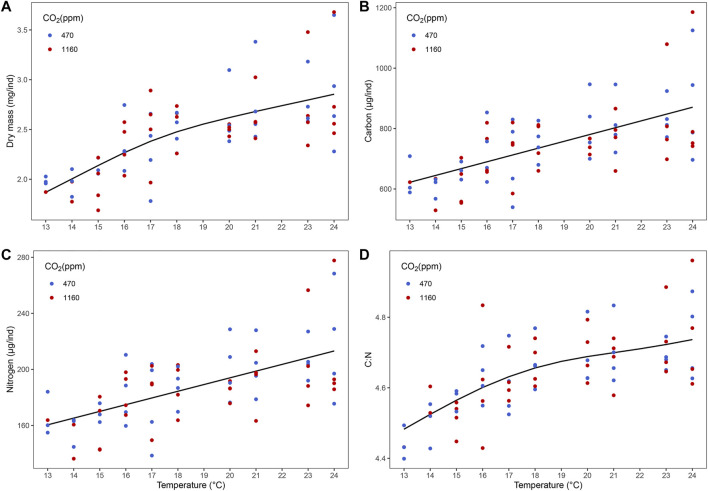
Effect of temperature on biomass of stage III *Homarus gammarus* larvae. **(A)** dry mass, **(B)** carbon content, **(C)** nitrogen content and **(D)** C:N was positively correlated with temperature. Each point represents the average response quantified in larvae originated from the same female. Curves correspond to smooths fitted with the best (general additive) model, obtained after backwards model selection.

**FIGURE 3:**
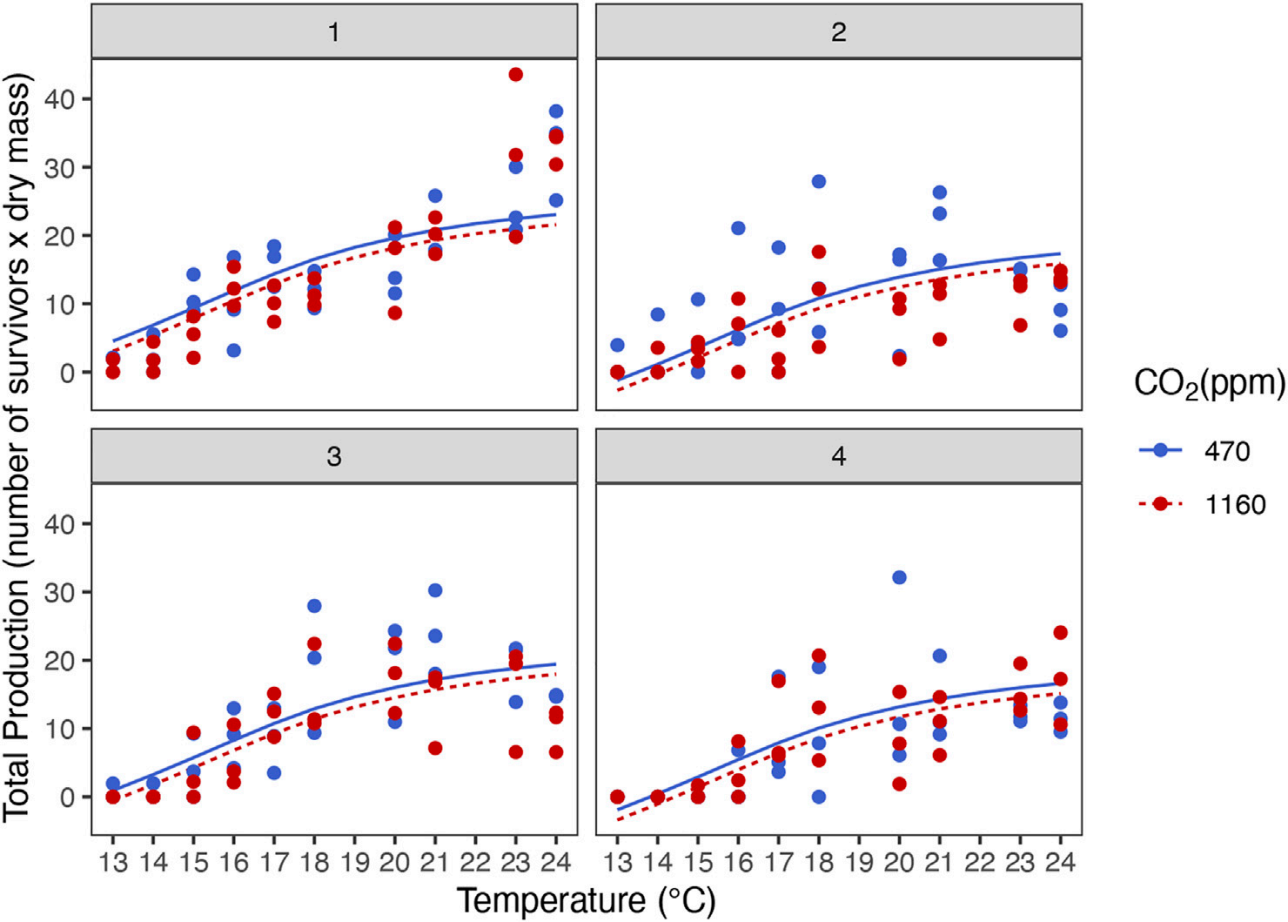
Additive effect of temperature and ocean acidification on total production of stage III *Homarus gammarus* larvae for the four hatches analyzed. Panels **1**–**4** respectively.

Best models retained temperature but not *p*CO_2_ as predictors (Figure 2 and Supplementary Table S4). Dry mass, carbon and nitrogen content, and C:N ratio increased with temperature. Instantaneous growth also increased with temperature but there was no evidence of an effect of *p*CO_2_ (Supplementary Figure S6). Temperature and *p*CO_2_ had an additive effect on total production (Figure 3). This result matches the trend and significance seen in the survival results.

### Morphological Measurements

**FIGURE 4:**
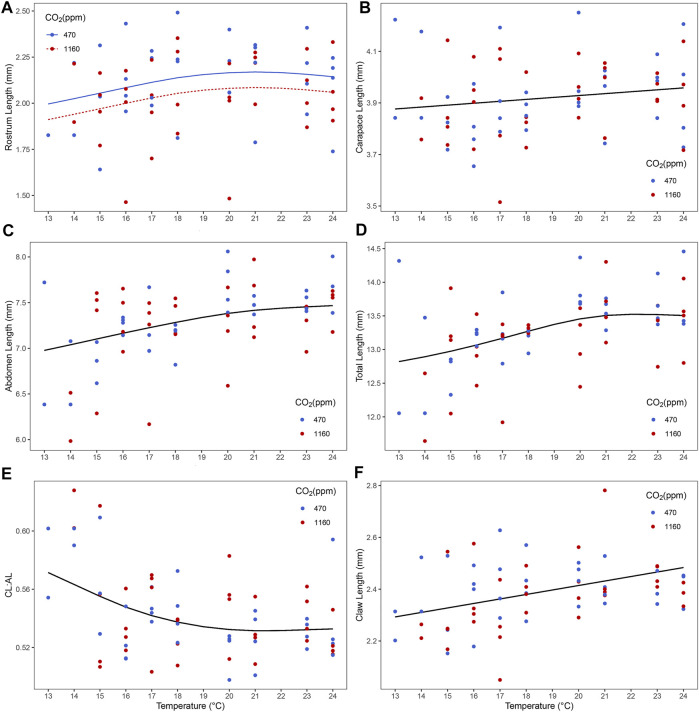
Effect of temperature and ocean acidification on size and morphology of stage III *Homarus gammarus* larvae. **(A)** ocean acidification and temperature effect on rostrum length; and temperature effect on **(B)** carapace length [CL], **(C)** abdomen length [AL], **(D)** total length, **(E)** CL: AL ratio and **(F)** claw length. Each point represents the average response quantified in larvae originated from the same female. Curves correspond to smooths fitted with the best (general additive) model, obtained after backwards model selection.

**FIGURE 5:**
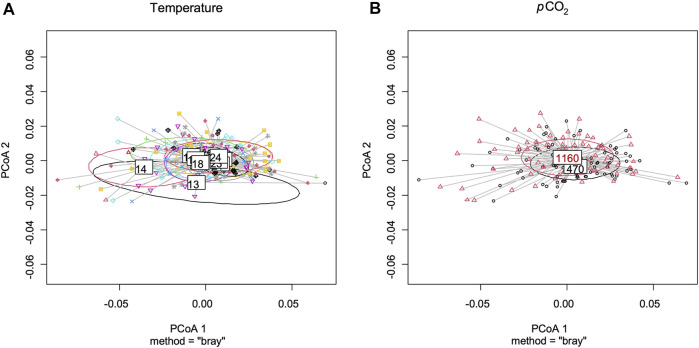
Results of principal coordinates analyses (PCoA) for morphological traits of stage III *Homarus gammarus* larvae. Plots were made using rostrum length, carapace length, abdomen length, total length and telson size. **(A)** comparison by temperatures (13–24°C), significant differences among temperatures. (PERMANOVA test: F1, 165 = 7.37, *p* = 0.003). **(B)** comparison by *p*CO_2_ concentrations, no significance differences among *p*CO_2_ concentrations.

Best models retained both *p*CO_2_ and temperature for predictors of rostrum length (Figure 4 and Supplementary Table S4). Lobster larvae in high *p*CO_2_ treatment had, on average, shorter rostrum length than those in moderate *p*CO_2_. Rostrum length (RL) increased with increasing temperature in both CO_2_ treatments. For the remaining variables, only temperature was retained in the best model (Supplementary Table S4). Carapace length (CL), abdomen length (AL), total length (TL) and claw size increased with temperature (Figure 4 and Supplementary Table S4). By contrast, the CL:AL ratio decreased with temperature (Figure 4 and Supplementary Table S3). Neither temperature nor *p*CO2 were retained as predictors for eye diameter size and telson length. Multivariate analysis using measured morphological characteristics (RL, CL, AL, TL and telson) did not give any significant morphological difference between larvae under moderate and high *p*CO_2_. Temperature had a significant effect on larval morphology in the colder temperatures (Figure 5; PERMANOVA test: F_1, 165_ = 7.37, *p* = 0.003).

### RMR Measurements and Antioxidant Enzyme Activity

**FIGURE 6:**
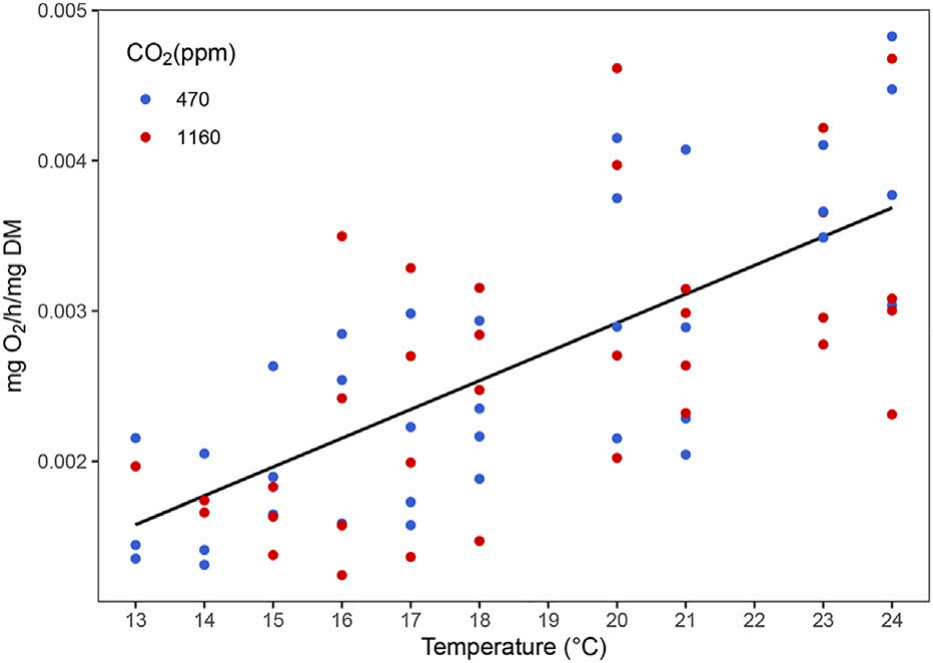
Effect of temperature on the routine metabolic rate of stage III *Homarus gammarus* larvae. Each point represents the average response quantified in larvae originated from the same female. Curves correspond to smooths fitted with the best (general additive) model, obtained after backwards model selection.

**FIGURE 7:**
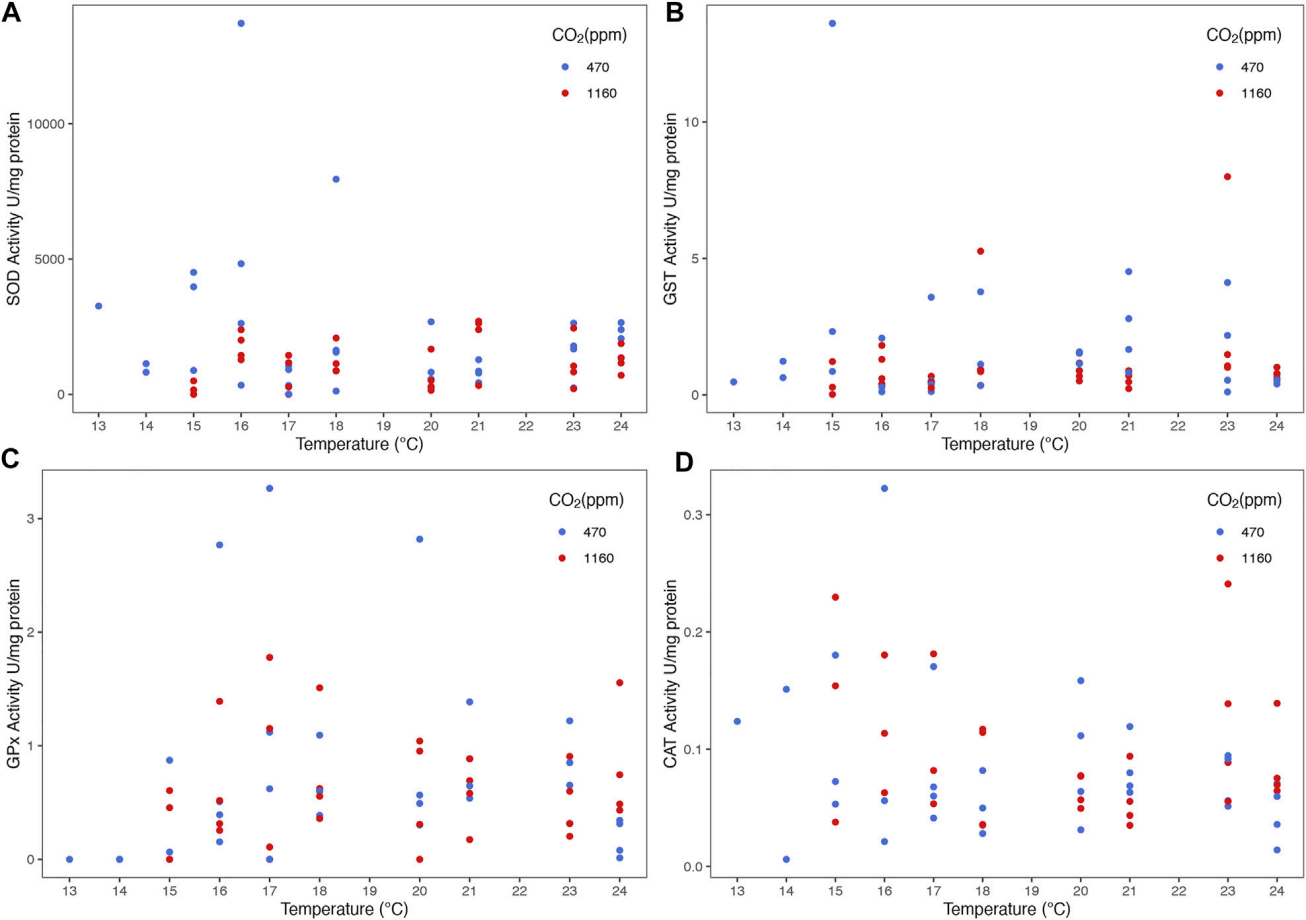
Effect of temperature and ocean acidification on antioxidant enzyme activities of stage III *Homarus gammarus* larvae. There was no effect of experimental conditions (temperature gradient and *p*CO_2_ levels) on antioxidant response: **(A)** SOD, **(B)** GST, **(C)** GPx and **(D)** CAT. Each point represents the average response quantified in larvae originated from the same female. Curves correspond to smooths fitted with the best (general additive) model, obtained after backwards model selection.

The routine metabolic rate (RMR) increased with temperature (Figure 6 and Supplementary Table S4). However, we did not find any evidence of an effect of OA (i.e. the best model contained only temperature as predictor). Best models did not retain temperature nor CO_2_ as predictors for variation in antioxidant activity of the enzymes SOD, GST, GPx and CAT (Figure 7).

## Discussion

Contrary to our expectations, we did not find any evidence of synergistic or interactive effects of temperature and *p*CO_2_ on any of the studied response variables. Ocean warming and acidification impacts can range from the highest level of sensitivity seen in the whole organism functioning, down to the cellular and molecular levels (Pörtner, 2008). We accordingly discuss our results from whole body functioning (i.e. survival, growth), to energy metabolism and finally to antioxidant responses. Our findings demonstrate that future high CO_2_ concentrations could have an impact on survival and morphology of lobster larvae. However, at the organizational levels analysed here, we did not detect physiological responses in lobster larvae. The latter will be discussed below.

### Survival

Our results suggest that near-future *p*CO_2_ conditions have a negative effect on lobster larvae survival to stage III. In lobster larvae, a bottleneck is expected in the transition between the last pelagic stage (stage III) and the benthic (stage IV); the stage III of *H. gammarus* is the one that accumulates most of the body mass leading to the stage IV (Torres et al., 2021). Such bottlenecks are important as life history transitions (Giménez, 2004; Pechenik, 2006; Marshall and Morgan, 2011). Our study clearly showed OA has the potential to increase mortality before reaching the transitional metamorphic stage (stage III to IV). In the wild, this could translate to an additional obstacle for successful recruitment. Similarly, reduced survival due to elevated *p*CO_2_ has been observed in early life stages in the European lobster (Small et al., 2016) and in the congeneric American lobster (Menu-Courey et al., 2019; Noisette et al., 2021). Moreover, this increase in mortality in early life stages of crustaceans exposed to ocean acidification has been noted in several other species: e.g. red king crab, *Paralithodes camtschaticus* (Long et al., 2013), edible crab, *Cancer pagurus* (Metzger et al., 2007) and the porcelain crab, *Pethrolisthes cinctipes* (Ceballos-Osuna et al., 2013). Previous studies on crustaceans and thermal stress revealed elevated *p*CO_2_ can narrow the thermal tolerance of the edible crab, *C. pagurus* and the spider crab, *H. araneus* (Metzger et al., 2007; Walther et al., 2009; Whiteley, 2011)*.* Our survival results provide no evidence of larvae reaching a temperature threshold or *pessimus* range on the warm side of our gradient with a maximum temperature (24°C) under high *p*CO_2_. However, on the cold side, the low number of lobster larvae that reached stage III at 13 and 14°C in both moderate and high *p*CO_2_ treatments suggest the *pessimus* survival limit is below 15°C. The lack of an interaction shows though that the limits were not affected by OA, in contrast to previous observations (stated above) and Pörtner’s (2008) predictions. The other significant driver for survival was temperature: higher temperatures resulted in higher survival in both moderate and high *p*CO_2_ treatments. We observed similarities with a study carried out in the same region (Helgoland) on the effect of climate warming on European lobster larvae (Schmalenbach and Franke 2010). Their results showed that optimal larval survival occurred within 16–22°C which largely matches our results; we also tested warmer temperatures (23°C and 24°C) where survival was even higher.

### Growth: Development Time, Biomass, Carbon and Nitrogen Content

**FIGURE 8:**
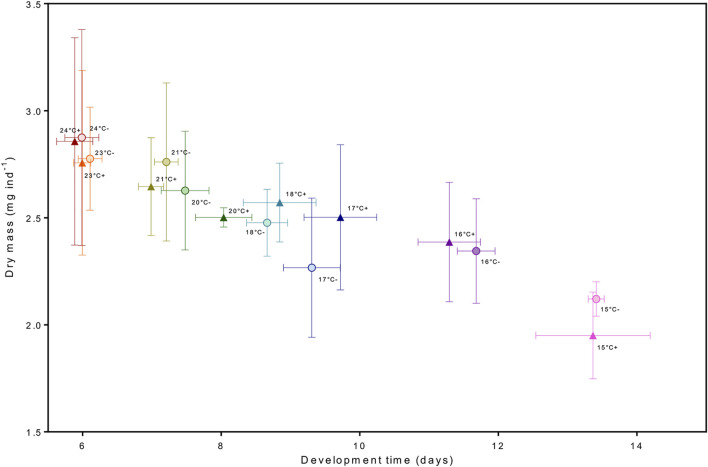
Integrated response between development time and drymass. The circle and (−) sign represent the moderate *p*CO_2_ treatment and the triangle and (+) sign the high *p*CO_2_ treatment.

There was no evidence of an effect of elevated *p*CO_2_ on development time from hatching to stage III. Our results are consistent with studies focusing on temperature only (Schmalenbach and Franke, 2010) as well as *p*CO_2_ and temperature (Arnold et al., 2009; Small et al., 2015; Waller et al., 2017) where *p*CO_2_ had no effect on lobster larval development rate. This led us to further enquire if there was possibly a trade-off between slower development rate under *p*CO_2_. For instance, at moderately low salinities, larval development of *H. gammarus* is extended, possibly as a way to minimize the negative effects on lipid and protein levels (Torres et al., 2021). Studies on the combined effect of food limitation and increased temperatures have analyzed the integrated response of dry mass and development under different temperatures (Torres and Giménez, 2020; Griffith et al., 2021) to provide insight if delayed development time could be a compensatory response to maintain body mass (and reserves) at stage. We investigated this integrated response, and our results show there was no trade-off between developing slower under elevated *p*CO_2_ conditions as larvae were reaching similar biomass when molting to stage III (Figure 8). Temperature alone was the principal driver in development rate, lobster larvae in warmer temperatures molted to stage III faster independently of *p*CO_2_ treatment. We did not find any evidence of effects of *p*CO_2_ on dry mass and elemental carbon (C) and (N) content, either; thus larvae grew to the thermal-dependent maximum body mass without any need of extending development. If present, the compensatory responses to increased *p*CO_2_ levels operated at a different level of organization, potentially at the intracellular level through acid-base balance mechanisms (Whiteley, 2011; Whiteley et al., 2018).

There was an increase in biomass and C and N content with increasing temperature, consistent with the increased survival. In crustacean larvae, carbon content (approximately >35% of biomass) is correlated with lipid content (a proxy for accumulation of reserves), while nitrogen (approximately 8–11% of biomass) reflects the protein content (Anger and Harms, 1990; Anger, 2001; Torres and Giménez, 2020). Biomass can also be divided into composition of proteins, lipids, chitins and free carbohydrates, making up >30%, <20%, <15%, and <5% respectively (Anger and Harms, 1990; Anger, 1998). However, chitin is mainly associated with cuticle formation and plays a minor role in the accumulation and utilization of energy reserves. Likewise, carbohydrates are stored as glycogen and for the synthesis of non-essential amino acids and thus have no significant influence on the C:N mass quotient (Anger and Harms, 1990).

The effect of temperature on biochemical composition in crustaceans has been studied in both field and controlled laboratory conditions. Field experiments showed that temperature changes, related to seasonal variation, can influence biochemical composition of decapod crustaceans (Buckup et al., 2008; Urzúa and Anger, 2013). Whereas laboratory experiments demonstrated that an increase in temperature led to an augmentation in lipid content in adult male whiteleg shrimps *(Litopenaeus vannamei)* (Perez-Velazquez et al., 2003) and a decrease in protein content in the northern shrimp larvae (*Pandalus borealis)* (Brillon et al., 2005). Our results are in line with previous results reporting higher lipid content in cherry shrimp (*Neocaridina heteropoda heteropoda)* at 24°C (Tropea et al., 2015). However, the same authors noted a decrease in lipid concentrations at 28°C and 32°C, which we did not see with our experimental design. We did not explore extremely high temperature where consumption rates of lipids (reflected in a decrease in carbon content) could have increased due to increased energy demands. More specifically, studies on American and European lobsters have shown varying results that are challenging to compare due to the difference in temperature treatments and life stages. A study done by Small et al. (2016) on juvenile European lobster acclimated at 10°C and 13°C showed carbon content decreased and nitrogen increased in the warmer temperature treatment. Another experiment done on stage I to stage IV European lobster larvae reared at 17°C and 21°C found no significant effects of elevated temperature on organic content (Small et al., 2015). Additionally, an experiment done on OA and warming on American lobster stage III larvae (Waller et al., 2017) showed higher C:N ratio at 19°C compared to 16°C. However, this difference was not seen in stage I, II or IV larvae. Our experiment similarly saw an increase in C:N ratio in stage III larvae at higher temperatures.

The high dry mass, and C and N content matches the energetic demands for rapid growth, accelerated cellular mechanisms and bigger size in warm temperatures. Because C:N ratios were lower towards the lower end of the temperature range, we hypothesize that such low temperatures limited the rate of accumulation of lipids more than that of proteins. Lower changes in proteins than in lipids has also been found when larvae are exposed to low salinity (Torres et al., 2002, 2021). In contrast, on the warmer side an increase in C:N ratio can be indicative of protein degradation due to high metabolism (Weiss et al., 2009). Thus, the decrease seen in C could be related to a degradation of lipids due to extra energetic requirements and a decrease in N could translate to a shortage in protein (Anger and Harms, 1990).

### Size and Morphology

Evidence of an effect of high *p*CO_2_ on size and morphology was found only for rostrum size, larvae exposed to high *p*CO2 showing shorter rostrum length than those in the high *p*CO_2_ treatment. The rostrum is the region which protects the eyes (Ingle, 1997), a reduced rostrum could potentially lead to eye damage; and proper eye-functioning is necessary for localizing prey and predators (Wahle, 1992). Consequently, we measured the eye diameter, as a proxy to assess if there was any correlation with nervous system underdevelopment (Letourneau, 1976; Laverack, 1988; Beltz and Sandeman, 2003) in elevated *p*CO_2._ Nevertheless, despite larvae having smaller rostrum in the high *p*CO_2_ there is no evidence that the eye size was influenced by high *p*CO2 or high temperatures. Additionally, we checked for “puffy” carapace, bent rostrum, and telson deformities, as seen in past studies of juvenile *H. gammarus* exposed to increased *p*CO_2_ at cold (12°C) and optimal temperatures (18°C) (Agnalt et al., 2013). Our results show only one case of a puffy carapace in larva under high *p*CO_2_ at 24°C. Lobster larvae reduced size under high *p*CO_2_ has been observed before (e.g. in carapace length (Keppel et al., 2012; Rato et al., 2017). Conversely, a study on *H. americanus* found a positive correlation between elevated *p*CO_2_ and carapace and abdomen length (Menu-Courey et al., 2019). We believe the effects on size observed in our study are not as strong as the study by Menu-Courey et al. (2019) and Noisette et al. (2021) possibly because the *p*CO_2_ gradient treatments used in those studies reached higher concentrations (1200, 2000 and 3000 µatm) than ours. Furthermore, our study only focused on larval stages and decapod larvae exoskeletons are unmineralized while those of benthic juveniles are partially calcified (Anger, 2001). This lack of calcification may be the reason why elevated *p*CO_2_ did not have a stronger impact on the size of the larvae’s different body parts.

### RMR and Antioxidant Enzyme Activity

Temperature is one of the most important factors influencing routine metabolic rates (RMR) in lobsters and other decapods (McLeese, 1964). Our results show that lobster larvae RMR increased linearly with increasing temperature, independent of *p*CO_2_ treatment. Our findings do not show an exponential relationship between RMR and temperature because this pattern is typically seen in standard and maximal metabolic rate measurements. Moreover, the results are highly dependent on the acclimation of animals, the larvae in our experiments were acclimated to a certain temperature and then respiration rate was measured at the same temperature. Exponential increase of oxygen consumption with temperature is usually seen and obtained with a different methodology (Schulte et al., 2011). A different approach where RMR is measured at acute temperatures independent of the temperature treatment larvae were acclimated to, could have help identify bottlenecks in cell functionality derived from the compensation costs provoked by combined higher temperature and *p*CO_2_ treatments. However, the design of our experiment and sample size did not allow for this kind of approach. The “Temperature Induced Metabolic Rate” method could be tested on lobster larvae in the future, as it is suitable for studying the effects of temperature on the metabolic capacities of non-constantly swimming organisms (Paschke et al., 2018). For this standardized method, the researcher is required to evaluate critical thermal maximum (CT max) and critical thermal minimum (CT min) to set the measurement temperature for high and low metabolic rates at each acclimation temperature to calculate an aerobic budget.

The higher RMR at warmer temperatures can be associated with faster development rate and larger size. In physiology, the cost of growing faster comes at the expense of an increase in feeding rates and thus swimming to catch food. These activities have been suggested to be energetically expensive in planktonic crustaceans (Morris et al., 1985). Our results are in line with previous studies on early life stages of *Homarus sp.* (Small et al., 2015; Waller et al., 2017; Menu-Courey et al., 2019), northern shrimps, *Pandalus borealis* (Arnberg et al., 2013)*,* and juvenile porcelain crabs, *P. cinctipides* (Carter et al., 2013 *),* where *p*CO_2_ did not significantly affect respiration rates. Significant oxidative stress responses would allow us to infer with more certainty on the optimal, *pejus* and p*essimus* ranges of lobster larvae. However, without evidence of significant antioxidant response fluctuations, it is difficult to separate *pejus* and *pessimus* ranges. From the oxygen consumption point of view and survival alone, the optimal range would be temperatures between 17–24°C as lobster were able to use their energy supply to maintain maximal physiological functions. Helgoland’s European lobster larvae appear to be quite tolerant to temperatures above those found in the German Bight (Schmalenbach and Franke, 2010).

On the cold side of our temperature gradients, our results show suboptimal temperatures under 15°C, expressed in low survival rates and low RMR. These temperatures are unusual for summertime in Helgoland when lobster larvae hatch (Schmalenbach and Franke, 2010). Nevertheless, temperatures recorded at the Helgoland long-term sampling indicate temperature increases are most noticeable during winter (Franke et al., 1999; Wiltshire et al., 2008). Experimental evidence shows winter warming (+3°C) can alter larval recruitment and result in lobster larvae hatching earlier, mid-April instead of mid-June. In the wild, lobster larvae could be faced with suboptimal temperatures that could cause the lengthening of development time in the pelagic stage, thus increasing the danger of mortality through predation (Schmalenbach and Franke, 2010).

Enzymatic antioxidant responses were measured for the first time in lobster larvae exposed to multiple environmental stressors. To date there is only one study by Rato et al. (2017) that analyzed the biochemical responses of *H. gammarus* under acidification alone and highlights the occurrence of oxidative stress. They found out lobster larvae under high *p*CO_2_ (710 µatm) had reduced SOD and higher DNA damage. Our study included variables not measured before under OA and thermal stress, such as the enzyme activity of GST, GPx and CAT. However, there was no evidence that OA and temperature had a negative impact on the antioxidant enzyme activity. We recognize deeper investigation at the molecular level (proteomic or transcriptomic) could reveal further information on the processes lobster larvae go through to cope with elevated *p*CO_2_ (Noisette et al., 2021)_._ For instance, Noisette et al. (2021) findings show elevated *p*CO_2_ (up to 3000 µatm) did not have an effect on larvae at a physiological level, however, there is evidence they underwent intensive metabolic reprogramming.

In conclusion, European lobster larvae demonstrated to be resilient to near future *p*CO_2_ concentrations at temperatures beyond 17–18°C, including higher temperatures than those experienced by the local population. Our results show larvae do not appear to have reached the critical temperatures or *pejus* range under the elevated temperatures tested (23–24°C). Raising the temperature even further and reaching the thermal limit of lobster larvae would have been interesting from a physiological point (e.g. higher antioxidant responses and compromised respiration). However, for the purposes of our research question, we wanted to understand how lobster larvae will cope with the predicted SSP5-8.5 scenario for 2100 in which SST will increase by 2-3°C. We observed no interactive effect of temperature and *p*CO_2_ on the measured variables; temperature was the greatest driver and there was an additive effect of *p*CO_2_ and temperature on survival and size. Examining the results from the perspective of different levels of biological organization, even though *p*CO_2_ did not elicit a response at the cellular level (i.e. enzyme activity) or physiological level (i.e development time to reach stage III); at the population level (survival) there were significant negative effects. We used total production (survival times biomass) as a way to integrate physiological and population responses, and it was evident there was an increase in mortality in larvae exposed to high *p*CO_2_ accompanied with lower biomass in the suboptimal temperatures (<15°C). Integrating physiological responses to environmental stressors and life history traits is key for species conservation strategies and stock enhancement management. Worst-case climate change scenarios could thus potentially have repercussion on ongoing restock efforts of endangered populations under recovery, like the European lobster population of Helgoland.

## Data Availability

The raw data supporting the conclusions of this article will be made available by the authors, without undue reservation.
