# Supplementary material for: European Lobster Larval Development and Fitness Under a Temperature Gradient and Ocean Acidification
Source: Front Physiol. 2022 Jul 14;13:809929. doi: 10.3389/fphys.2022.809929 (PMC9333128; doi:10.3389/fphys.2022.809929)
Supplement: Supplementary file 1 [file DataSheet1.docx]

Supplementary Material

# Supplementary Tables

| **Supplementary Table 1.** Visualization of the thermal gradient incubator setup (10 x 6) including daily water parameters measured during the duration of the all the experimental runs (mean$\pm$sd) for each temperature and CO_2_ treatment combination. The circles represent one beaker containing 15 (hatch 1) or 12 (hatches 2, 3, and 4) lobster larvae each. Each treatment had three replicates (beakers). | | | | | | | | | | |
| --- | --- | --- | --- | --- | --- | --- | --- | --- | --- | --- |
| **Moderate *p*CO_2_** | | | | | | | | | | |
|  | 1 | 2 | 3 | 4 | 5 | 6 | 7 | 8 | 9 | 10 |
| A |  |  |  |  |  |  |  |  |  |  |
| B |  |  |  |  |  |  |  |  |  |  |
| C |  |  |  |  |  |  |  |  |  |  |
| T (ºC) | 12.6$\pm$0.5 | 13.6$\pm$0.5 | 15.1$\pm$0.3 | 16.0$\pm$0.4 | 17.3$\pm$0.4 | 18.1$\pm$0.1 | 19.9$\pm$0.4 | 20.9$\pm$0.4 | 22.9$\pm$0.3 | 23.9$\pm$0.5 |
| Salinity | 32.5$\pm$1.1 | 32.3$\pm$0.9 | 32.6$\pm$1.1 | 32.5$\pm$1.0 | 32.4$\pm$0.9 | 32.4$\pm$0.9 | 32.7$\pm$0.9 | 32.6$\pm$0.9 | 32.4$\pm$0.8 | 32.3$\pm$1.0 |
| pH | 8.0$\pm$0.1 | 8.0$\pm$0.1 | 8.0$\pm$0.1 | 8.0$\pm$0.1 | 8.0$\pm$0.1 | 8.0$\pm$0.1 | 8.0$\pm$0.1 | 8.0$\pm$0.1 | 8.0$\pm$0.1 | 8.0$\pm$0.1 |
| **High *p*CO_2_** | | | | | | | | | | |
| D |  |  |  |  |  |  |  |  |  |  |
| E |  |  |  |  |  |  |  |  |  |  |
| F |  |  |  |  |  |  |  |  |  |  |
| T (ºC) | 12.8$\pm$0.5 | 13.8$\pm$0.4 | 15.2$\pm$0.3 | 16.0$\pm$0.4 | 17.5$\pm$0.4 | 18.2$\pm$0.4 | 19.8$\pm$0.3 | 21.0$\pm$0.3 | 22.9$\pm$0.4 | 23.8$\pm$0.4 |
| Salinity | 32.3$\pm$1.0 | 32.3$\pm$1.0 | 32.2$\pm$1.0 | 32.1$\pm$0.9 | 32.2$\pm$0.9 | 32.2$\pm$0.8 | 32.3$\pm$0.9 | 32.1$\pm$0.8 | 32.2$\pm$0.7 | 32.2$\pm$0.9 |
| pH | 7.8$\pm$0.1 | 7.7$\pm$0.1 | 7.7$\pm$0.1 | 7.8$\pm$0.1 | 7.8$\pm$0.1 | 7.8$\pm$0.1 | 7.8$\pm$0.1 | 7.8$\pm$0.1 | 7.8$\pm$0.1 | 7.8$\pm$0.1 |

| **Supplementary Table 2.** Seawater carbonate chemistry of CO_2_ treatments *Homarus gammarus* larvae were exposed during the experiments. | | | | | | | |
| --- | --- | --- | --- | --- | --- | --- | --- |
| Row &  Treatment | T (^o^C) | pH | TA (μmol/kgSW) | ^1^TCO_2_ (μmol/kgSW) | ^1^pCO_2_ (μatm) | ^1^HCO3− (μmol/kgSW) | ^1^CO32− (μmol/kgSW) |
| 1. Moderate *p*CO_2_ | 13.0 ± 0.6 | 8.12 ± 0.02 | 2320 ± 29 | 2148 ± 29 | 456 ± 23 | 1999 ± 30 | 131 ± 6 |
| 1. High *p*CO_2_ | 13.0 ± 0.6 | 7.75 ± 0.03 | 2294 ± 26 | 2251 ± 28 | 1147 ± 88 | 2145 ± 28 | 61 ± 5 |
| 2. Moderate *p*CO_2_ | 13.7 ± 0.4 | 8.14 ± 0.04 | 2314 ± 33 | 2125 ± 42 | 431 ± 49 | 1967 ± 46 | 141 ± 8 |
| 2. High *p*CO_2_ | 13.8 ± 0.5 | 7.76 ± 0.03 | 2306 ± 44 | 2260 ± 44 | 1156 ± 81 | 2152 ± 42 | 63 ± 3 |
| 3. Moderate *p*CO_2_ | 15.0 ± 0.3 | 8.11 ± 0.02 | 2327 ± 12 | 2143 ± 5 | 473 ± 26 | 1986 ± 9 | 139 ± 7 |
| 3. High *p*CO_2_ | 15.1 ± 0.3 | 7.75 ± 0.03 | 2306 ± 35 | 2255 ± 33 | 1171 ± 82 | 2145 ± 31 | 66 ± 5 |
| 4. Moderate *p*CO_2_ | 15.9 ± 0.2 | 8.11 ± 0.01 | 2335 ± 39 | 2150 ± 33 | 482 ± 16 | 1992 ± 29 | 141 ± 7 |
| 4. High *p*CO_2_ | 15.8 ± 0.3 | 7.76 ± 0.02 | 2307 ± 27 | 2251 ± 29 | 1157 ± 54 | 2140 ± 28 | 68 ± 2 |
| 5. Moderate *p*CO_2_ | 17.2 ± 0.1 | 8.12 ± 0.04 | 2273 ± 84 | 2077 ± 62 | 463 ± 37 | 1915 ± 49 | 146 ± 17 |
| 5. High *p*CO_2_ | 17.3 ± 0.4 | 7.78 ± 0.03 | 2322 ± 16 | 2256 ± 14 | 1139 ± 75 | 2142 ± 15 | 74 ± 4 |
| 6. Moderate *p*CO_2_ | 17.9 ± 0.2 | 8.11 ± 0.03 | 2327 ± 26 | 2123 ± 36 | 483 ± 40 | 1955 ± 41 | 152 ± 8 |
| 6. High *p*CO_2_ | 18.0 ± 0.4 | 7.79 ± 0.02 | 2373 ± 101 | 2302 ± 97 | 1148 ± 58 | 2184 ± 92 | 78 ± 6 |
| 7. Moderate *p*CO_2_ | 20.0 ± 0.5 | 8.12 ± 0.03 | 2336 ± 9 | 2120 ± 18 | 487 ± 39 | 1944 ± 25 | 161 ± 9 |
| 7. High *p*CO_2_ | 19.7 ± 0.2 | 7.76 ± 0.02 | 2341 ± 29 | 2269 ± 24 | 1208 ± 53 | 2151 ± 21 | 78 ± 4 |
| 8. Moderate *p*CO_2_ | 20.8 ± 0.3 | 8.12 ± 0.02 | 2351 ± 32 | 2130 ± 25 | 490 ± 13 | 1948 ± 20 | 164 ± 6 |
| 8. High *p*CO_2_ | 20.9 ± 0.3 | 7.80 ± 0.03 | 2306 ± 26 | 2221 ± 17 | 1103 ± 74 | 2102 ± 13 | 84 ± 7 |
| 9. Moderate *p*CO_2_ | 22.8 ± 0.2 | 8.15 ± 0.06 | 2358 ± 34 | 2101 ± 59 | 454 ± 77 | 1898 ± 74 | 189 ± 18 |
| 9. High *p*CO_2_ | 23.0 ± 0.3 | 7.79 ± 0.03 | 2384 ± 27 | 2289 ± 21 | 1177 ± 71 | 2160 ± 18 | 93 ± 5 |
| 10. Moderate *p*CO_2_ | 24.1 ± 0.4 | 8.15 ± 0.01 | 2342 ± 51 | 2084 ± 55 | 454 ± 28 | 1880 ± 55 | 191 ± 21 |
| 10. High *p*CO_2_ | 23.8 ± 0.3 | 7.81 ± 0.02 | 2391 ± 32 | 2287 ± 37 | 1149 ± 75 | 2154 ± 37 | 98 ± 4 |
| *TA values were calculated at the beginning and end of each experimental run to ensure conditions were constant; based on salinity (32 psu), temperature, pH and total alkalinity (TA) using CO2SYS software. Values are represented as mean* $\pm$ *standard deviation.^1^Parameters calculated using CO_2_SYS Excel Macro software (Pierrot et al., 2006) with constants provided by Mehrbach et al., (1973) refitted by Dickson and Millero (1987) and KHSO_4_ constants from Dickson (1990).* | | | | | | | |

| **Supplementary Table 3.** Biomass, carbon and nitrogen content measured in freshly hatched larvae | | | |
| --- | --- | --- | --- |
| Hatch | Dry mass (mg) | Nitrogen (µg/ind) | Carbon (µg/ind) |
| 1 | 1.6 | 135.9 | 545.2 |
| 1 | 1.7 | 148.0 | 594.9 |
| 1 | 1.6 | 139.0 | 549.8 |
| 1 | 1.7 | 139.9 | 560.4 |
| 1 | 1.7 | 143.1 | 568.2 |
| 1 | 1.6 | 133.1 | 526.5 |
| 1 | 1.6 | 138.4 | 544.9 |
| 1 | 1.7 | 152.2 | 608.5 |
| 1 | 1.7 | 146.4 | 581.9 |
| 1 | 1.6 | 137.4 | 549.2 |
| 1 | 1.7 | 143.5 | 574.2 |
| 1 | 1.7 | 142.3 | 570.1 |
| 1 | 1.7 | 142.0 | 559.9 |
| 1 | 1.7 | 148.5 | 584.8 |
| 2 | 1.4 | 119.3 | 465.7 |
| 2 | 1.6 | 132.9 | 522.9 |
| 2 | 1.4 | 121.2 | 475.0 |
| 2 | 1.2 | 98.9 | 387.4 |
| 2 | 1.5 | 127.2 | 500.5 |
| 2 | 1.2 | 103.4 | 404.1 |
| 2 | 1.4 | 126.1 | 494.2 |
| 2 | 1.5 | 129.2 | 522.8 |
| 2 | 1.4 | 121.4 | 475.0 |
| 2 | 1.5 | 126.7 | 514.9 |
| 2 | 1.5 | 126.9 | 507.5 |
| 2 | 1.5 | 133.4 | 525.8 |
| 2 | 1.3 | 106.4 | 422.5 |
| 2 | 1.5 | 134.1 | 528.8 |
| 2 | 1.3 | 110.5 | 430.2 |
| 3 | 1.6 | 137.7 | 551.9 |
| 3 | 1.7 | 149.7 | 590.2 |
| 3 | 1.6 | 139.4 | 549.0 |
| 3 | 1.6 | 140.8 | 555.3 |
| 3 | 1.5 | 129.4 | 504.0 |
| 3 | 1.6 | 140.6 | 554.5 |
| 3 | 1.7 | 147.4 | 578.4 |
| 3 | 1.7 | 140.4 | 545.8 |
| 4 | 1.5 | 129.8 | 548.4 |
| 4 | 1.7 | 139.7 | 590.7 |
| 4 | 1.5 | 128.5 | 571.0 |
| 4 | 1.6 | 134.0 | 578.7 |
| 4 | 1.7 | 138.3 | 582.9 |
| 4 | 1.6 | 131.7 | 564.1 |
| 4 | 1.6 | 128.5 | 571.0 |
| 4 | 1.6 | 128.3 | 541.2 |
| 4 | 1.5 | 138.3 | 582.9 |
| 4 | 1.6 | 129.1 | 538.0 |
| 4 | 1.7 | 141.3 | 599.6 |
| 4 | 1.7 | 134.5 | 577.6 |
| 4 | 1.6 | 138.7 | 578.9 |
| 4 | 1.7 | 135.0 | 576.9 |
| 4 | 1.7 | 139.2 | 591.3 |

| **Supplementary Table 4.** Results of the statistical models applied for analysis of ocean acidification and temperature changes in *Homarus gammarus* larvae. temp= temperature, s=smooth function in RStudio. Codes: *** when p-value is value < 0.00001; ** when p-value < 0.001; * when p-value < 0.05. | | | | | | | | | |
| --- | --- | --- | --- | --- | --- | --- | --- | --- | --- |
| **Parameter** | **Model** | **Fixed effects** | **Random effect** | **Terms** | **Estimate** | **Std. error** | **t value** | **Pr(>\|t\|)** |  |
| Survival | s(temp) + CO2 + s(hatch) | temp+CO_2_ | hatch | intercept | 33.29 | 2.76 | 12.09 | <2e-16 | *** |
|  |  |  |  | CO_2_ | -5.37 | 2.03 | -2.64 | 0.0088 | ** |
|  |  |  |  | **Smooth Terms** | **edf** | **Ref.df** | **F** | **p-value** |  |
|  |  |  |  | temp | 3.38 | 4.16 | 65.66 | <2e-16 | *** |
|  |  |  |  | hatch | 2.53 | 3 | 5.36 | 0.0004 | *** |
| Development | s(temp) + s(hatch) | temp | hatch | **Terms** | **Estimate** | **Std. error** | **t value** | **Pr(>\|t\|)** |  |
|  |  |  |  | intercept | 9.27 | 0.33 | 28.45 | <2e-16 | *** |
|  |  |  |  | **smooth terms** | **edf** | **Ref.df** | **F** | **p-value** |  |
|  |  |  |  | temp | 5.92 | 7.06 | 208.65 | <2e-16 | *** |
|  |  |  |  | hatch | 2.78 | 3 | 12.22 | <2e-16 | *** |
| RL | s(temp) + CO2 + s(hatch) | temp+CO_2_ | hatch | **Terms** | **Estimate** | **Std. error** | **t value** | **Pr(>\|t\|)** |  |
|  |  |  |  | intercept | 2.13 | 0.1 | 22.36 | <2e-16 | *** |
|  |  |  |  | CO_2_ | -0.08 | 0.03 | -2.85 | 0.005 | ** |
|  |  |  |  | **smooth terms** | **edf** | **Ref.df** | **F** | **p-value** |  |
|  |  |  |  | temp | 2.18 | 2.72 | 3.18 | 0.03 | * |
|  |  |  |  | hatch | 2.92 | 3 | 34.96 | <2e-16 | *** |
| CL | temp + s(hatch) | temp | hatch | **Terms** | **Estimate** | **Std. error** | **t value** | **Pr(>\|t\|)** |  |
|  |  |  |  | intercept | 3.78 | 0.09 | 41.1 | <2e-16 | *** |
|  |  |  |  | temp | 0.007 | 0.003 | 1.98 | 0.0496 | * |
|  |  |  |  | **smooth terms** | **edf** | **Ref.df** | **F** | **p-value** |  |
|  |  |  |  | hatch | 2.86 | 3 | 21.31 | <2e-16 | *** |
| AL | s(temp) + s(hatch) | temp | hatch | **Terms** | **Estimate** | **Std. error** | **t value** | **Pr(>\|t\|)** |  |
|  |  |  |  | intercept | 7.32 | 0.1 | 75.36 | <2e-16 | *** |
|  |  |  |  | **smooth terms** | **edf** | **Ref.df** | **F** | **p-value** |  |
|  |  |  |  | temp | 1.70 | 2.12 | 5.7 | 0.003 | ** |
|  |  |  |  | hatch | 2.59 | 3 | 4.78 | 0.0009 | *** |
| TL | s(temp) + s(hatch) | temp | hatch | **Terms** | **Estimate** | **Std. error** | **t value** | **Pr(>\|t\|)** |  |
|  |  |  |  | intercept | 13.32 | 0.12 | 111.1 | <2e-16 | *** |
|  |  |  |  | **smooth terms** | **edf** | **Ref.df** | **F** | **p-value** |  |
|  |  |  |  | temp | 2.21 | 2.76 | 6.28 | 0.0006 | *** |
|  |  |  |  | hatch | 2.45 | 3 | 4.77 | 0.0007 | *** |
| CL:AL | s(temp) + s(hatch) | temp | hatch | **Terms** | **Estimate** | **Std. error** | **t value** | **Pr(>\|t\|)** |  |
|  |  |  |  | intercept | 0.54 | 0.007 | 77.76 | <2e-16 | *** |
|  |  |  |  | **smooth terms** | **edf** | **Ref.df** | **F** | **p-value** |  |
|  |  |  |  | temp | 2.16 | 2.7 | 4.11 | 0.013 | * |
|  |  |  |  | hatch | 2.43 | 3 | 4.49 | 0.001 | ** |
| Claw | s(temp) + s(hatch) | temp | hatch | **Terms** | **Estimate** | **Std. error** | **t value** | **Pr(>\|t\|)** |  |
|  |  |  |  | intercept | 2.40 | 0.040 | 60.07 | <2e-16 | *** |
|  |  |  |  | **smooth terms** | **edf** | **Ref.df** | **F** | **p-value** |  |
|  |  |  |  | temp | 1.00 | 1 | 17.03 | 5.8e-05 | *** |
|  |  |  |  | hatch | 2.67 | 3 | 8.35 | 9.5e-05 | *** |
| Respiration Rate | temp only | temp | hatch | **Terms** | **Estimate** | **Std. error** | **t value** | **Pr(>\|t\|)** |  |
|  |  |  |  | intercept | -0.0009 | 0.0001 | -1.73 | 0.085 |  |
|  |  |  |  | temp | 0.0002 | 0.00003 | 7.51 | 3.05E-12 | *** |
| C:N | s(temp) + s(hatch) | temp | hatch | **Terms** | **Estimate** | **Std. error** | **t value** | **Pr(>\|t\|)** |  |
|  |  |  |  | intercept | 4.66 | 0.03 | 147.2 | <2e-16 | *** |
|  |  |  |  | **smooth terms** | **edf** | **Ref.df** | **F** | **p-value** |  |
|  |  |  |  | temp | 2.70 | 3.36 | 31.49 | <2e-16 | *** |
|  |  |  |  | hatch | 2.87 | 3 | 22.43 | <2e-16 | *** |
|  |  |  |  | **Terms** | **Estimate** | **Std. error** | **t value** | **Pr(>\|t\|)** |  |
|  |  |  |  | intercept | 2.51 | 0.10 | 24.65 | <2e-16 | *** |
| Dry mass | s(temp) + s(hatch) | temp | hatch | **smooth terms** | **edf** | **Ref.df** | **F** | **p-value** |  |
|  |  |  |  | temp | 2.33 | 2.9 | 36.37 | <2e-16 | *** |
|  |  |  |  | hatch | 2.80 | 3.0 | 14.99 | <2e-16 | *** |
|  |  |  |  | **Terms** | **Estimate** | **Std. error** | **t value** | **Pr(>\|t\|)** |  |
|  |  |  |  | intercept | 760.8 | 38.36 | 19.83 | <2e-16 | *** |
| Carbon | s(temp) + s(hatch) | temp | hatch | **smooth terms** | **edf** | **Ref.df** | **F** | **p-value** |  |
|  |  |  |  | temp | 1.0 | 1.0 | 96.70 | <2e-16 | *** |
|  |  |  |  | hatch | 2.88 | 3.0 | 26.99 | <2e-16 | *** |
|  |  |  |  | **Terms** | **Estimate** | **Std. error** | **t value** | **Pr(>\|t\|)** |  |
|  |  |  |  | intercept | 189.87 | 8.56 | 22.18 | <2e-16 | *** |
| Nitrogen | s(temp) + s(hatch) | temp | hatch | **smooth terms** | **edf** | **Ref.df** | **F** | **p-value** |  |
|  |  |  |  | temp | 1.0 | 1.0 | 87.81 | <2e-16 | *** |
|  |  |  |  | hatch | 2.89 | 3.0 | 27.17 | <2e-16 | *** |
| SOD | hatch only |  | hatch | **Terms** | **Estimate** | **Std. error** | **t value** | **Pr(>\|t\|)** |  |
|  |  |  |  | intercept | 1640.2 | 369.4 | 4.44 | 1.74e-05 | *** |
|  |  |  |  | **smooth terms** | **edf** | **Ref.df** | **F** | **p-value** |  |
|  |  |  |  | hatch | 2.0 | 3.0 | 2.26 | 0.02 | * |
| GST | null |  |  | **Terms** | **Value** | **Std. error** | **t value** | **p-value** |  |
|  |  |  |  | intercept | 1.5 | 0.25 | 6.03 | 0 |  |
| GPx | null |  |  | **Terms** | **Value** | **Std. error** | **t value** | **p-value** |  |
|  |  |  |  | intercept | 0.69 | 0.07 | 9.73 | 0 |  |
| CAT | null |  |  | **Terms** | **Value** | **Std. error** | **t value** | **p-value** |  |
|  |  |  |  | intercept | 0.88 | 0.01 | 11.86 | 0 |  |

**2. Supplementary Figures**

**Results by Hatch**

**Supplementary Figure 1**. Effect of temperature and ocean acidification on the survival and development time of *Homarus gammarus* larvae to stage III for the four hatches analyzed (panels 1 to 4 respectively). **(A)** ocean acidification and temperature influenced survival, **(B)** temperature effect on development time.

**
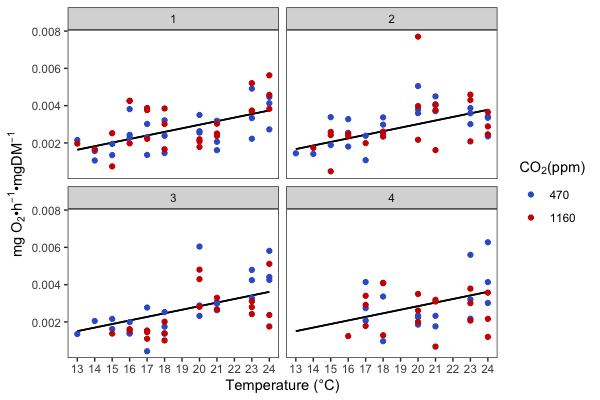
**

**Supplementary Figure 2**. Effect of temperature on the routine metabolic rate of stage III *Homarus gammarus* larvae for the four hatches analyzed (panels 1 to 4 respectively).

**Supplementary Figure 3**. Effect of temperature on biomass of stage III *Homarus gammarus* larvae for the four hatches analyzed (panels 1 to 4 respectively). **(A)** C:N ratio, **(B)** dry mass, **(C)** carbon content and **(D)** nitrogen content was positively correlated with temperature.

**Supplementary Figure 4.** Effect of temperature and ocean acidification on size and morphology of stage III *Homarus gammarus* larvae for the four hatches analyzed (panels 1 to 4 respectively). **(A)** ocean acidification and temperature effect on rostrum length, and temperature effect on **(B)** carapace length [CL], **(C)** abdomen length [AL], **(D)** total length, **(E)** CL: AL ratio and **(F)** claw length.

**Supplementary Figure 5.** There was no effect of experimental conditions (temperature gradient and *p*CO_2_ levels) on antioxidant response. **(A)** SOD, **(B)** GST, **(C)** GPx and **(D)** CAT

**
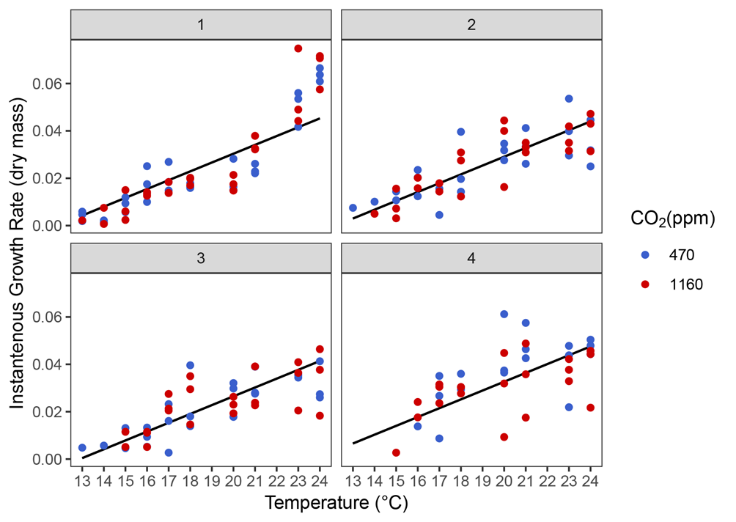
**

**Supplementary Figure 6.** Temperature effect on instantaneous growth of stage III *Homarus gammarus* larvae for the four hatches analyzed (panels 1 to 4 respectively).
